# Inflammasome-Induced Osmotic Pressure and the Mechanical Mechanisms Underlying Astrocytic Swelling and Membrane Blebbing in Pyroptosis

**DOI:** 10.3389/fimmu.2021.688674

**Published:** 2021-07-07

**Authors:** Zihui Zheng, Tingting Wang, Jiahui Chen, Huimin Qiu, Chencheng Zhang, Weizhen Liu, Simiao Qin, Jilai Tian, Jun Guo

**Affiliations:** ^1^ School of Medicine & Holistic Integrative Medicine, Nanjing University of Chinese Medicine, Nanjing, China; ^2^ Key Laboratory of Drug Target and Drug for Degenerative Disease, Nanjing University of Chinese Medicine, Nanjing, China

**Keywords:** pyroptotic astrocyte, inflammasome protein nanoparticles, protein nanoparticle-induced osmotic pressure, cell swelling and membrane blebbing, intermediate filament tension

## Abstract

Cell swelling and membrane blebbing are characteristic of pyroptosis. In the present study, we explored the role of intracellular tension activity in the deformation of pyroptotic astrocytes. Protein nanoparticle-induced osmotic pressure (PN-OP) was found to be involved in cell swelling and membrane blebbing in pyroptotic astrocytes, and was associated closely with inflammasome production and cytoskeleton depolymerization. However, accumulation of protein nanoparticles seemed not to be absolutely required for pyroptotic permeabilization in response to cytoskeleton depolymerization. Gasdermin D activation was observed to be involved in modification of typical pyroptotic features through inflammasome-induced OP upregulation and calcium increment. Blockage of nonselective ion pores can inhibit permeabilization, but not inflammasome production and ion influx in pyroptotic astrocytes. The results suggested that the inflammasomes, as protein nanoparticles, are involved in PN-OP upregulation and control the typical features of pyroptotic astrocytes.

## Introduction

Neuroinflammation is one of the most common pathologies of the central nervous system (CNS) and innate immune response in neural tissue to restrain infection and eliminate pathogens (1). Lines of evidence suggest that neuroinflammation is involved in the pathological process of glial pyroptosis induced by sepsis (2–4). Astrocytes are abundant type of glia cells, essential for regulating ions and neurotransmitters in the CNS milieu. As astrocyte pyroptosis involves in the pathological progression of the nervous system diseases, the cytopathic mechanisms need to be further clarified.

Astrocytic pyroptosis displays membrane blebbing, cell swelling, and further promotes pore formation on the astrocyte membrane, which exacerbates membrane permeabilization (5–7). Alterations of cell shape depend on cytoskeleton remodeling, which provides the cytoskeletal tractive forces against the outward osmotic pressure (OP) across the membrane (8–10). Our previous study suggested that the production of protein nanoparticles (PNs) in astrocytes upregulates cell osmolarity significantly by adsorbing cations and driving the influx of ions and water, thereby promoting cell swelling (8).

Inflammasome production and caspase activation act as the upstream stimulation events in astrocytic pyroptosis. In the inflammasome assembly, nucleotide binding oligomerization domain like receptor family pyrin domain protein 3 (NLRP3) is activated *via* protein phosphatase 2 phosphatase activator (PP2A)-mediated dephosphorylation of the pyrin domain (PYD) and JUN N-terminal kinase 1 (JNK1)-mediated phosphorylation of the S198 site between the PYD and nucleotide-binding domain (NACHT). After NLRP3 combines with apoptosis-associated spok-like protein (ASC) to form oligomer, protein tyrosine kinase 2 beta (PYK2)-mediated phosphorylation of the caspase recruitment domain (CARD) facilitates the recruitment of pro-caspase 1 to achieve the active inflammasome complex (11, 12). Active caspase-1 cleaves gasdermin D (GSDMD) to obtain GSDMD-CT (C terminal fragment) and GSDMD-NT (N terminal fragment), the latter one of which locates on the cell membrane to facilitate the formation of membrane pores (13, 14). The resultant open of the nonselective channels can induce ions influx (7, 15); however, how to regulate cell swelling and rupture is still unclear in astrocytic pyroptosis. During the inflammatory process, inflammasome production might increase the amount of intracellular PNs in pyroptotic cells. Alternatively, caspase-1-mediated cofilin dephosphorylations lead to microfilament (MF) depolymerization to produce actin (16, 17). Whether intracellular PNs elicited by pyroptosis are involved in the OP increment and astrocytic deformation remains to be clarified.

Several studies have assessed the calcium ion signal in the process of pyroptosis, and it is widely accepted that the calcium pathway is an upstream event regulating the occurrence of pyroptosis. NLRP3 inflammasome is activated by potassium efflux and calcium influx, and then the active caspase-1 mediates pyroptosis and inflammation (18–22). The amplification of Ca^2+^ entry signal occurs through Ca^2+^ release from the endoplasmic reticulum (ER) channels during pyroptosis, including the inositol 1,4,5-trisphosphate receptor (IP3R) and the ryanodine receptor (RyR) (23).

In the present study, we aimed to determine the effects of PN-OP in the process of astrocyte pyroptosis using fluorescence resonance energy transfer (FRET)-based intermediate filament (IF) tension probes and measuring the cytoplasmic OP. Activation of the neuroinflammatory pathway facilitated inflammasome production, which resulted in OP increment and in turn promoted cell swelling and blebbing, thereby regulating the cell volume and pyroptosis.

## Materials and Methods

### Reagents, Antibodies and Small Interfering RNA (siRNA) Design

4-Hydroxytamoxifen was purchased from Aladdin (Shanghai, China). Jasplakinolide, taxol and caffeine were obtained from Abcam (Cambridge, UK). 2-Aminoethyl diphenylborinate, dantrolene, Z-VAD-FMK, SP600125, lipopolysaccharide (LPS) and nigericin were purchased from MedChemExpress (Monmouth Junction, NJ, USA). MCC950 were obtained from CSNpharm (Chicago, IL, USA). PEG8000 and PF431396 were obtained from Beyotime Biotechnology (Shanghai, China). Cantharidin was purchased from Sigma-Aldrich (Saint Louis, MO, USA). Rabbit anti-caspase-1, rabbit anti-ASC and rabbit anti-NLRP3 antibodies were obtained from Proteintech (Chicago, IL, USA). Mouse anti-α-tubulin antibody was from Boster (BM1452, Wuhan, China). Mouse anti-β-actin was purchased from Cell Signaling Technology (Danvers, MA, USA). The siRNA targeting *ASC* and *CASPASE-1* was constructed by GenePhrama (Shanghai, China). The Flag-Gsdmd-NT, Flag-Gsdmd-CT plasmid were purchased from Addgene (Watertown, MA, USA).

### Cell Culture and Transfection

The human glioblastoma cell line U87 was obtained by the American Type Culture Collection (ATCC, Manassas, USA). Cell were cultured at 37°C, 5% CO_2_ with complete Dulbecco’s modified Eagle’s Medium (Invitrogen, New York, USA) containing 10% fetal bovine serum (Invitrogen), 100 units/ml penicillin (Invitrogen) and 100 μg/ml streptomycin. Transfections were carried out using 2 μg of *ASC* or *CASPASE-1* siRNA (GenePhrama, Shanghai, China) mixed with 2 μl of Lipofectamine 2000™ (Invitrogen) in 200 μl of Opti-MEM medium, incubated for 20 min. 800 μl of 1% medium was next added and incubated for 12 hours.

### Animals

The principles of laboratory animal care were followed and all procedures were conducted according to the guidelines established by the National Institutes of Health. The study protocol was approved by the Research Animal Care Committee of Nanjing University of Chinese Medicine. Adult male C57BL/6 mice (15-20 g, six weeks old) were obtained from the Model Animal Research Center of Nanjing University of Chinese Medicine.

### Measurement of the Cytoplasmic OP and the Count Rate of Protein Particles

When cells reached 90% confluence, medium supplemented with special drugs replaced the old medium. The supernatant was obtained by digestion, centrifugation (12000×g, 4°C), ultrasonication (Sonics and Materials, Connecticut, CT, USA) and re-centrifugation. The osmotic pressure and distribution of cytoplasmic nanoparticles were determined using freezing point osmometer and Nanosight NS300 (Malvern Analytical, Malvern, UK).

### Probe Construction and Transfection

Tension sensors were produced using the NovoRec PCR Seamless Cloning Kit and restriction enzyme cloning techniques according to previous reports (24–27).We constructed fluorescent sensors with circularly permutated cpVenus and cpCerulean [cpVenus–7aa–cpCerulean (cpstFRET)]. The vimentin probe comprised PCMV-Vimentin-cpCerulean-7aa-cpVenus (cpstFRET)-Vimentin. Plasmids were extracted from single colonies and purified according to the manufacturer’s instructions.

### cpstFRET Analyses

The dipole angle between donor/eCFP and acceptor/eYFP determined the effectiveness of FRET. Cells were imaged using a confocal microscope (SP8; Leica, Wetzlar, Germany) equipped with a ×63 oil-immersion objective lens. The donor and acceptor were tested by argon lasers at 458 nm and 514 nm, respectively. The CFP/FRET ratios were calculated using the equation 1/E = cerulean donor/venus acceptor.

### FRET-AB and FRAP Analyses

We applied LAS AF Application Wizard v1.7.0 (Leica) for detailed analyses of probes, including live cell acceptor photobleaching FRET (FRET-AB) experiments and fluorescence recovery after photobleaching (FRAP) experiments. The acceptor of the whole cell was bleached and then we calculated the efficiency of FRET. The constructed recovery curve was used to estimate probe activities.

### Calcium and Chloride Fluorescence Imaging

Cells were loaded with Fluo-4 AM (Ca^2+^ imaging) or N-6-methoxyquinolyl acetoethyl ester (MQAE, Cl^-^ imaging) fluorescent probe. Fluo-4 fluorescence was detected using Thunder Imager (SP8; Leica) at excitation and emission wavelengths of 494 nm and 516 nm. MQAE fluorescence was detected at excitation and emission wavelengths of 355nm and 460nm, respectively. The increase of intracellular Cl^-^ levels led to the decrease of MQAE fluorescence value instead.

### Immunohistochemistry (IHC) Staining and Analysis

Mouse brain tissues were stained using antibodies for caspase-1. Semi-quantitative analysis of the IHC images was conducted using Image J, in which integral optical density (IOD) and the area data were collected. Then, the average optical density (AOD) was calculated as IOD/area, which represented the staining intensity.

### Live-Cell Imaging

To examine cell-death morphology, cells were treated as indicated in 96-well plates. Static bright-field images of pyroptotic cells were captured using IncuCyte Zoom at room temperature and processed using Image J software. For staining, 1 μg/mL propidium iodide (PI) dye (Annexin V-FITC/PI double staining apoptosis detection Kit, Nanjing Jiancheng Bioengineering Institute, Nanjing, China) or Sytox Green dye (Beyotime Biotechnology, Shanghai, China) was added immediately after the stimulations, as per manufacturer’s instructions.

### Lactate Dehydrogenase Release (LDH) Test and Caspase-1 Activity Assay

LDH release was measured using the LDH release quantification cytotoxicity Assay Kit (Beyotime Biotechnology; C0016) in accordance with the manufacturer’s instructions. Caspase-1 activity was measured using a caspase-1 activity assay kit (Beyotime Biotechnology; C1101) based on the cleavage of substrate YVAD-AFC, according to the manufacturer’s instructions.

### RT-qPCR

The cDNA synthesis was performed using 1 μg mRNA and HiScript II RT SuperMix for qPCR (Vazyme, Nanjing, China). The SYBR Green Master Mix (YEASEN, Shanghai, China) was used in the fluorescent quantification of PCR. β-actin was used to normalize mRNA levels.

### Immunofluorescence

Cells were washed with phosphate-buffered saline (PBS), fixed in 4% paraformaldehyde solution and washed again. The cell membrane was permeabilized in 0.1% Triton X-100 at 4°C for 10 min. Cells were blocked with PBS containing 4% serum for 30 min and then incubated with primary antibodies overnight at 4°C. After washing with PBS, cells were incubated with the secondary antibodies in the dark. DAPI was used to stain the cell nuclei. Immunofluorescent staining was examined using a Leica confocal microscope (DMi8; Leica).

### Statistical Analyses

The FRET ratio in each subcellular region was measured for each cell and then averaged over multiple cells. Images were processed and pseudo-colored using the 16-color map of Image J. All data were presented as mean ± SEM. One way ANOVA with the least significant difference test was adopted to determine statistical significance. Each experiment was repeated at least three times, >10 cells were imaged, and each condition was analyzed. P value were determined by t-test. Ns (not significant), p > 0.05, *p < 0.05, **p < 0.01, ***p < 0.001.

More detailed materials and methods can be found in the **Supplementary Materials**.

## Results

### Cytoskeleton Stabilization Partly Inhibited Cell Swelling and Blebbing, but Did Not Alter Permeabilization

**Figure 1:**
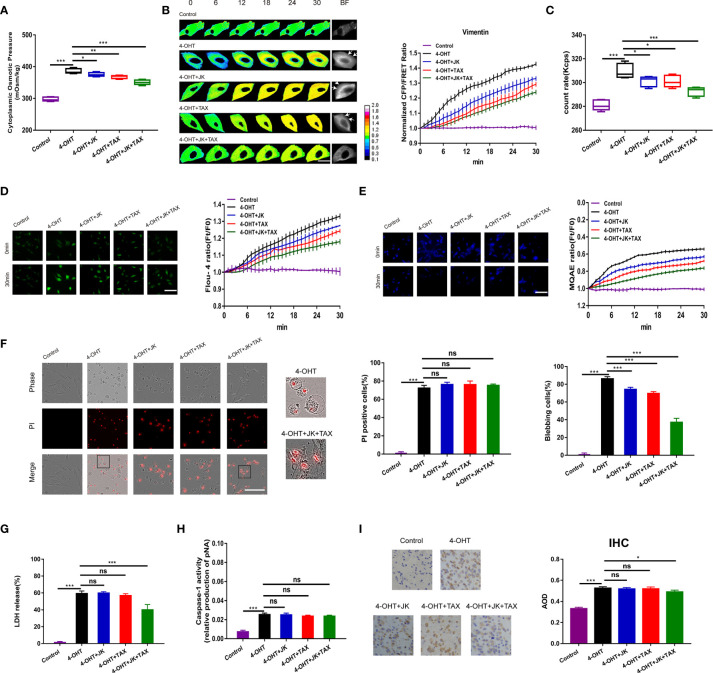
MF and MT stabilization antagonized cell swelling but did not alter membrane permeability. **(A)** The cytoplasmic OP values of U87 cells were measured using a freezing point osmometer under 4-OHT treatment (5 μg/ml), co-treatment of 4-OHT with jasplakinolide (JK, 10 μM), taxol (TAX, 10 μM), both agents, and the isotonic control. **(B)** Representative images and mean values of normalized CFP/FRET ratios of Vimentin tension under the different treatments. Arrows indicate the membrane blebbing regions. The calibration bar was set from 0.1 to 2.0. Scale bar, 10 μm. **(C)** The count rate of PNs in U87 cells. **(D)** Calcium imaging micrographs and traces of relative Fluo-4 fluorescence intensity (Ft/F0) of cells. Scale bar, 100 μm. **(E)** Chloridion imaging micrographs and traces of relative MQAE fluorescence intensity (Ft/F0) of cells. The increase of intracellular Cl^-^ levels led to the decrease of MQAE fluorescence value instead. Scale bar, 100 μm. **(F)** Representative time-lapse images of U87 cells subjected to different treatments. Cell membrane permeabilization was monitored by PI uptake (red fluorescence). The bar charts represent the percentage of PI positive cells and blebbing cells, respectively. Scale bar, 100 μm. **(G)** LDH release and **(H)** caspase-1 activity of cells under the different treatments. **(I)** IHC analysis of caspase-1 expression in brain section of the sepsis mouse models. The cytoplasm was stained with the caspase-1 antibody (brown). Semiquantitative analysis of the IHC staining by the average optical density (AOD) among the groups. Mean of ≥ 3 experiments ± SEM. Values marked with asterisks are significantly different, as determined using a *t*-test. ns, not significant.

Pyroptosis causes cell death, accompanied by dramatic changes in cellular morphology. The pyroptotic astrocyte model was constructed using the 4-hydroxytamoxifen (4-OHT (5 µg/ml) treatment (9). OP is the predominant factor that controls cell morphology and is regulated by intracellular PNs (8). Indeed, the cytoplasmic OP values measured by the freezing point osmometer were increased dramatically after 4-OHT treatment (**Figure 1A**). Considering that IF tension is closely associated with OP alteration (8), we could effectively evaluate IF tension using the FRET-based Vimentin tension probes (**Figure S1**). The results showed the significantly increased IF tension under 4-OHT stimulus (**Figure 1B**); and the count rate of PNs increased dramatically in response to 4-OHT (**Figure 1C**). Meanwhile, the other model of pyroptosis (induced by lipopolysaccharide-nigericin, LPS-NIG) showed similar results (**Figures S2A, B**). These data suggested the production of abundant PNs in astrocytes and increased OP across the cell membrane occurred during the pyroptosis process. To further explore the relationship between cell osmolarity and intracellular ion contents, Fluo-4 AM and MQAE fluorescent probes were used to detect intracellular Ca^2+^ and Cl^-^ levels, respectively. Both the Ca^2+^ and Cl^-^ levels were upregulated significantly after 4-OHT stimulus (**Figures 1D, E**), implying that the free ion levels in the cells were associated closely with PN-OP.

Given the increase in IF tension and the intracellular PN level in response to 4‑OHT stimulus, we hypothesized that cytoskeletal depolymerization to produce PNs occurred in the process of pyroptosis. Jasplakinolide (JK, 10 µM), and taxol (TAX, 10 µM) were employed to stabilize microfilament (MF) and microtubule (MT), respectively (28–30). Treatment with JK, TAX, or both partly attenuated 4-OHT-induced upregulation of the cytoplasmic OP, IF tension, intracellular PN number, and calcium and chloride ion levels (**Figures 1A–E**); while showed little impact on the LPS-NIG pyroptotic model (**Figure S2**). These results suggested that MF and MT stabilization partly reduced the 4-OHT-induced OP alteration, accompanied by decreased levels of intracellular ions. Regarding the cell morphology, the pyroptotic astrocytes showed cell swelling and blebbing (a high percentage of blebbing cells), and increased membrane permeabilization (a high percentage of propidium iodide (PI) positive cells, **Figure 1F**). Notably, JK and TAX co-treatments presented the additive effects due to the accumulatively decreased OP and PN amount, contributing to more obvious reduction in membrane blebbing compared with the individual treatment. However, MF/MT stabilizers did not alter the permeabilization, showing insignificant differences of PI positive cells (**Figure 1F**). JK and TAX co-treatments prevented the LDH release stimulated by 4-OHT (**Figure 1G**), implying that LDH release imperfectly represented the occurrence of pyroptosis (31). Alternatively, caspase‑1 activity, as a factor used to determine pyroptotic cells, appeared to increase after 4-OHT stimulation; while JK, TAX, or both had no effect on this process (**Figure 1H**). Similar results were obtained in the LPS-NIG model of pyroptotic astrocytes (**Figures S2C–E**).

To further analyze the variation in pyroptosis progression *in vivo*, intraperitoneal injection of 4-OHT into the mouse model was performed and caspase-1 expression was assessed using immunohistochemical (IHC) staining of the pyroptotic section in the brain. The average optical density (AOD) representing caspase-1 expression in the 4-OHT group was increased significantly; whereas JK and TAX co-treatments slightly reduced the AOD (**Figure 1I**), which was in accordance with the astrocyte results. These data indicated that a cytoskeletal stabilization-induced decrease in PNs attenuated cell swelling and blebbing, but failed to suppress pyroptotic permeabilization. These results suggested that the increase in PN-OP caused by cytoskeleton depolymerization contributed to astrocyte swelling and blebbing, which seems to be accompanied with the pyroptosis process, but not the absolute factor of permeabilization.

### ASC-Induced Inflammasome Assembly Regulated PN-OP Is Involved in Pyroptosis

**Figure 2:**
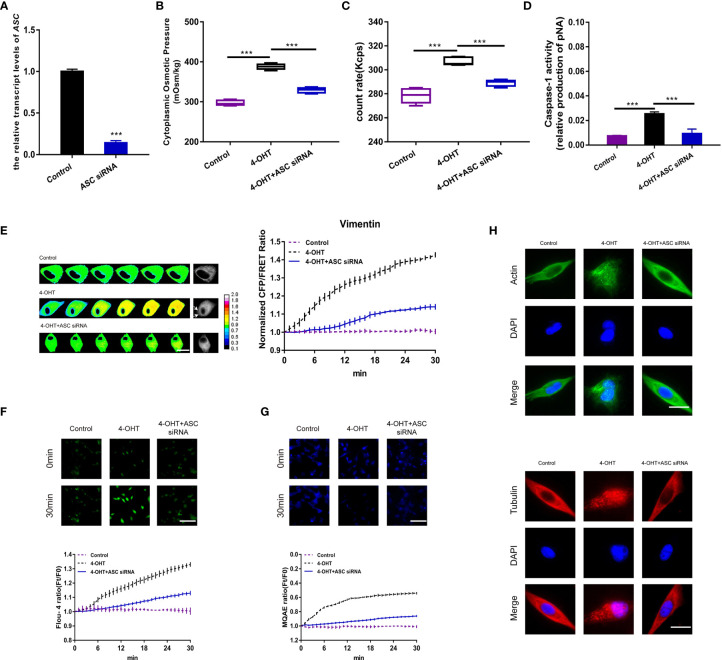
ASC-induced inflammasome assembly regulated PN-OP. **(A)** RT-qPCR was used to measure the relative transcript level of *ASC* after transient transfection with the *ASC* siRNA. **(B)** The cytoplasmic OP values of U87 cells were measured using a freezing point osmometer under co-treatment of 4-OHT with the *ASC* siRNA. **(C)** The count rate of PNs. **(D)** Caspase-1 activity of cells. **(E)** Representative images and mean values of normalized CFP/FRET ratios of Vimentin tension under the different treatments. Arrows indicate the membrane blebbing regions. The calibration bar was set from 0.1 to 2.0. Scale bar, 10 μm. **(F)** Calcium imaging micrographs and traces of relative Fluo-4 fluorescence intensity (Ft/F0) of cells. **(G)** Chloridion imaging micrographs and traces of relative MQAE fluorescence intensity (Ft/F0) of cells. The increase of intracellular Cl^-^ levels led to the decrease of MQAE fluorescence value instead. **(H)** Monolayers of U87 cells were subjected to 4-OHT treatment alone, or co-treatment with 4-OHT and the *ASC* siRNA, then stained with β-actin (FITC) or α-tubulin (TRITC). The images were generated from the fluorescence inverted microscope after immunofluorescence staining. Mean of ≥ 3 experiments ± SEM. Values marked with asterisks are significantly different, as determined using a *t*-test.

To further investigate the PN-OP effects derived from NLRP3 PNs in pyroptosis, ASC, as a crucial component of the NLRP3 inflammasome (19), was effectively knocked-down *via* transfection of an *ASC* siRNA into astrocytes (**Figure 2A**). When NLRP3 assembly was blocked, the increase in intracellular OP, PN amount, caspase-1 activity, IF tension, and permeabilization induced by 4-OH were reduced dramatically (**Figures 2B–E** and **Figure S4**). In addition, the intracellular Ca^2+^ and Cl^-^ levels decreased after *ASC* siRNA transfection (**Figures 2F, G**). Compared with the 4-OHT treatment, the lack of ASC rescued the MF and MT cleavage (**Figure 2H**), suggesting that blockade of NLRP3 assembly might inhibit the downstream caspase-1-mediated cleavage of MF and MT. Overall, these data indicated that inflammasomes served as intracellular PNs to increase the OP and facilitate astrocyte pyroptosis.

### NLRP3 Inflammasomes Are Involved in PN-OP Regulation of Pyroptosis

**Figure 3:**
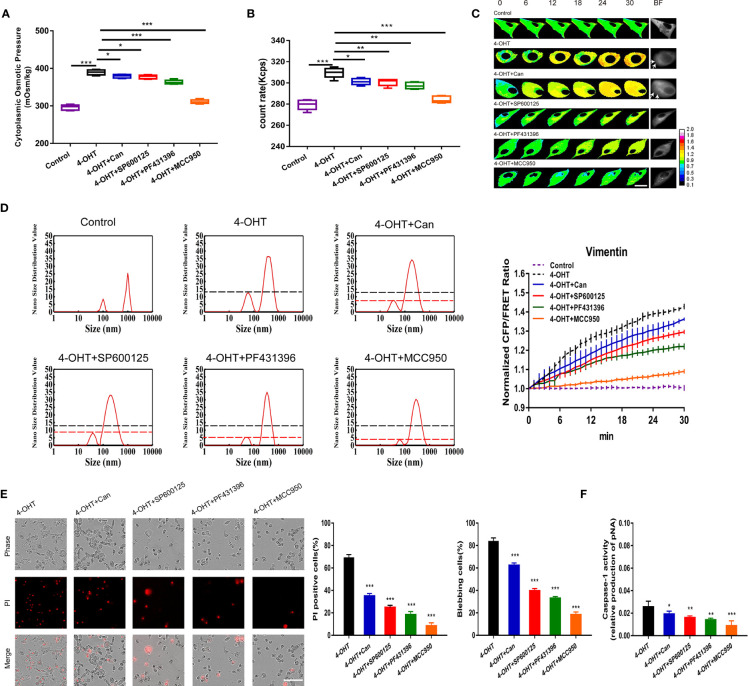
Blockade of NLRP3 assembly and activation downregulated PN-OP and inhibited pyroptosis. **(A)** The cytoplasmic OP values of U87 cells were measured using a freezing point osmometer under co-treatments of 4-OHT with cantharidin (10 μM), SP600125 (20 μM), PF431396 (15 μM), or MCC950 (1 µM), respectively. **(B)** The count rate of PNs in U87 cells. **(C)** Representative images and mean values of normalized CFP/FRET ratios of Vimentin tension under the different treatments. Arrows indicate the membrane blebbing regions. **(D)** Nano size distribution of protein granules in the cytoplasm. **(E)** Representative time‑lapse images of U87 cells. Cell membrane integrity was monitored by PI uptake (red fluorescence). The bar charts represent cells showing PI positive and blebbing. **(F)** Caspase-1 activity of cells. Mean of ≥ 3 experiments ± SEM. Values marked with asterisks are significantly different, as determined using a *t*-test.

**Figure 4:**
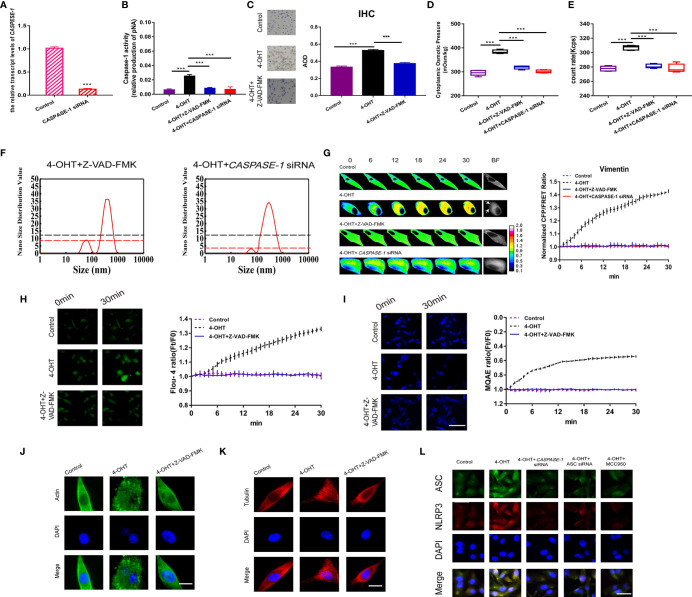
Caspase-1 inhibitor decreased PN-OP and prevented pyroptosis. **(A)** RT-qPCR was used to measure the relative transcript level of *CASPASE-1* after transient transfection with the *CASPASE-1* siRNA. **(B)** Z‑VAD-FMK (20 μM) treatment or *CASPASE-1* siRNA transfection of U87 cells inhibited the caspase-1 activity. **(C)** IHC analysis of caspase-1 expression in brain section of the sepsis mouse models. The cytoplasm was stained with the caspase-1 antibody (brown). Semiquantitative analysis of the IHC staining by comparison of AOD among the groups. **(D)** The cytoplasmic OP values of U87 cells were measured using a freezing point osmometer. **(E)** The count rate of PNs in U87 cells. **(F)** Nano size distribution value of protein granules in the cytoplasm. Black dash line represents value of the 50-100 nm peak in the 4-OHT group. **(G)** Representative images and mean values of normalized CFP/FRET ratios of Vimentin tension. Arrows indicate the membrane blebbing regions. **(H)** Calcium imaging micrographs and traces of relative Fluo-4 fluorescence intensity (Ft/F0) of cells. **(I)** Chloridion imaging micrographs and traces of relative MQAE fluorescence intensity (Ft/F0) of cells. **(J, K)** Monolayers of U87 cells were stained for β-actin (FITC), α-tubulin (TRITC). **(L)** Immunofluorescence of ASC and NLRP3 in U87 cells using antibodies. The images were generated from the fluorescence inverted microscope after immunofluorescence staining. Mean of ≥ 3 experiments ± SEM. Values marked with asterisks are significantly different, as determined using a *t*-test.

The NLRP3 inflammasome, as polyprotein complex, has been demonstrated to be involved in pyroptosis (32). We hypothesized that NLRP3 inflammasomes could serve as PNs to regulate PN-OP during pyroptosis. Protein phosphatase 2a (PP2A) inhibitor (cantharidin, 10 µM), JNK1 inhibitor (SP600125, 20 µM), pky2 inhibitor (PF431396, 15 µM) and the NLRP3-selective inhibitor (MCC950, 1 µM) were used individually to block the assembly and activation of NLRP3 oligomers through (de)phosphorylation or NLRP3-induced ASC oligomerization (12, 33, 34). Immunofluorescence of ASC and NLRP3 showed the increased levels of NLRP3 inflammasome during pyroptosis, and MCC950 effectively attenuated (**Figure 4L**). Compared with the 4-OHT group, three pan inhibitors partly reduced the cytoplasmic OP and the number of PNs; (**Figures 3A, B**), similar results were obtained in the LPS-NIG pyroptosis model (**Figures S3A, B**). Particularly, MCC950 decreased potently the 4-OHT-induced OP and the PN amount almost to the control levels (**Figures 3A, B**). In the control group, the size distribution of the cytoplasmic PN particles was around 100 nm and 1000 nm. However, 4-OHT treatment produced smaller PNs around 50-100 nm and 100-1000 nm; and NLRP3 inhibitors reduced the number of PNs around the 30-100 nm size distribution (**Figure 3D**). In addition, these inhibitors decreased the 4-OHT-mediated tension enhancement (**Figure 3C**), and effectively relieved permeabilization, cell blebbing and caspase-1 activity; of these, MCC950 showed the highest inhibitory efficacy (**Figures 3E, F** and **Figures S3C–E**, **S4**). Based on these data, we speculated that the morphological changes of astrocytes during pyroptosis were closely associated with the PN-OP increase, caused by NLRP3 inflammasomes acting as PNs.

### Caspase-1 Is Involved in the Inflammatory Pathways That Facilitated the Production of Massive PNs, Regulating Pyroptosis Deformation

Caspase-1, involved in the inflammatory signaling pathway, is activated by NLRP3 inflammasomes and then cleaves GSDMD to produce GSDMD-NT, which facilitates pore-forming and induces permeabilization (35). To determine the roles of inflammasome PNs in pyroptosis occurrence, firstly, caspase-1 activity was inhibited by the pan inhibitor (Z-VAD-FMK, 20 µM) and *CASPASE-1* siRNA in astrocytes (**Figures 4A, B**). *In vivo*, caspase-1 inhibition rescued 4-OHT-induced pyroptosis, as shown by the recovered AOD representing caspase-1 expression using IHC staining in brain sections (**Figure 4C**). Regarding to the increased cytoplasmic OP and numbers of PNs in the 4-OHT or LPS-NIG pyroptosis model, they were reduced almost to the control levels after treatment of caspase-1 inhibitor or *CASPASE-1* siRNA (**Figures 4D, E** and **Figures S3A, B**). Capase-1 inhibition reduced the number of PNs around 50-100 nm (**Figure 4F**). Meanwhile, the 4-OHT-induced upregulation of IF tension, intracellular Ca^2+^ and Cl^-^ contents, and permeabilization were drastically reduced under Z-VAD-FMK treatment or *CASPASE-1* siRNA, even reaching the same levels as the control group (**Figures 4G–I** and **Figure S4**). Caspase-1 plays a role in cleaving MF and MT in the pyroptotic process, resulting in the MF/MT structure turning into punctiform granules, which was recovered using the caspase-1 inhibition (**Figures 4J, K**). Furthermore, caspase-1 inhibition reduced the levels of NLRP3 inflammasome, shown in the immunofluorescent levels of ASC, NLRP3 and merge (**Figure 4L**). These data suggested that caspase-1 exerts feedback regulation in the generation of NLRP3 inflammasomes, accompanied by controlling the intracellular Ca^2+^ levels in astrocytes.

### Block of the Non-Selective Ion Channels Played Little Role in PN-OP Regulation

**Figure 5:**
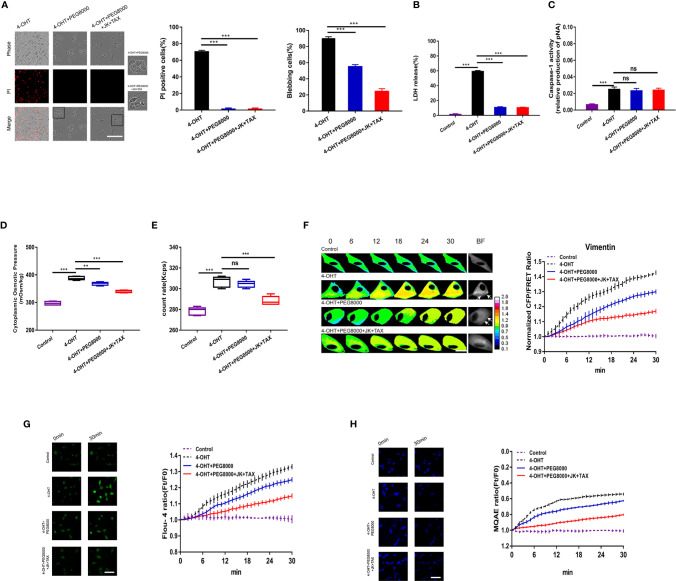
Blockade of non-selective pores using PEG8000 prevented membrane permeabilization but did not affect cell swelling. **(A)** Representative time-lapse images of U87 cells under 4-OHT treatment, and co-treatments of 4-OHT with PEG8000, and the additional MF/MT stabilizers (JK and TAX). Cell membrane integrity was monitored by PI uptake (red fluorescence). The bar charts represent PI positive and blebbing cells. **(B)** LDH release and **(C)** caspase-1 activity of cells. **(D)** The cytoplasmic OP values. **(E)** The count rate of PNs in U87 cells. **(F)** Representative images and mean values of normalized CFP/FRET ratios of Vimentin tension under the different treatments. Arrows indicate the membrane blebbing regions. **(G)** Calcium imaging micrographs and traces of relative Fluo-4 fluorescence intensity (Ft/F0) of cells. **(H)** Chloridion imaging micrographs and traces of relative MQAE fluorescence intensity (Ft/F0) of cells. Mean of ≥ 3 experiments ± SEM. Values marked with asterisks are significantly different, as determined using a *t*-test. ns, not significant.

The formation of non-selective pores on the cell membrane appears to accompany the occurrence of pyroptosis (36, 37); however, little attention has been paid to its roles in cell osmotic effects. Polyethylene glycol (PEG8000) was used to block the inflow of non-selective ions across the plasma membrane (15). In the present study, 4‑OHT combined with either PEG8000 or additional cytoskeletal stabilizers inhibited cell permeabilization, and decreased the proportion of blebbing cells and LDH release (**Figures 5A, B**), which was consistent with the LPS-NIG model data (**Figures S5C–E**). Both models demonstrated that blockade of membrane pores altered the morphology of pyroptotic cells. In addition, PEG8000 could partly attenuate the 4‑OHT or LPS-NIG-induced upregulation of the cytoplasmic OP (**Figure 5D** and **Figure S5A**) and IF tension (**Figure 5F**), and slightly decreased the intracellular Ca^2+^ and Cl^-^ levels (**Figures 5G, H**). However, blockade of non-selective pores could not interfere with caspase-1 activity or intracellular PN production (**Figures 5C, E** and **Figure S5B**). These results suggested that blockade of non-selective ion channels could not prevent the production of NLRP3 PNs, and the upregulation of PN-OP in the pyroptosis process might not be absolutely caused by an influx of non-selective ions.

Furthermore, compared with the 4-OHT-PEG8000 group, the additional treatment with cytoskeleton stabilizers partly decreased the proportion of blebbing cells (**Figure 5A**), the cytoplasmic OP, the number of PNs, IF tension, and the Ca^2+^ and Cl^-^ levels (**Figures 5D–H**); but did not change membrane permeabilization, LDH release, or caspase-1 activity (**Figures 5A–C**). This was consistent with the above data (**Figure 1**), confirming that cytoskeleton stabilization could suppress osmolarity‑dependent astrocyte swelling, rather than membrane permeabilization.

### Calcium Ions Released From the Endoplasmic Reticulum Regulated Pyroptosis *via* PN Production

**Figure 6:**
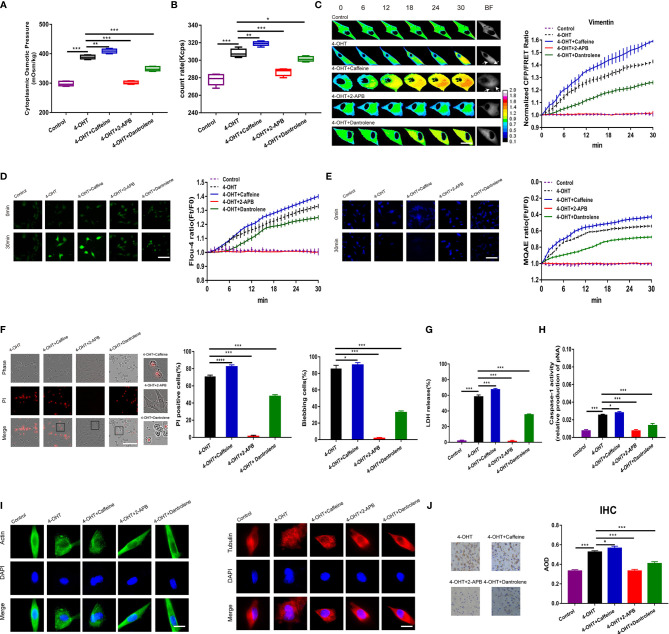
Calcium ions released from the endoplasmic reticulum regulated pyroptosis. **(A)** The cytoplasmic OP values of U87 cells were measured using a freezing point osmometer under the isotonic control, 4-OHT treatment, and co-treatments of 4-OHT with caffeine (2 mM), 2-APB (100 μM), and dantrolene (20 μM). **(B)** The count rate of PNs in U87 cells. **(C)** Representative images and mean values of normalized CFP/FRET ratios of Vimentin tension under the different treatments. Arrows indicate the membrane blebbing regions. **(D)** Calcium imaging micrographs and traces of relative Fluo-4 fluorescence intensity (Ft/F0) of cells. **(E)** Chloridion imaging micrographs and traces of relative MQAE fluorescence intensity (Ft/F0) of cells. **(F)** Representative time‑lapse images of U87 cells subjected to different treatments. Cell membrane integrity was monitored by PI uptake (red fluorescence). The bar charts represent percentage of cells with PI positivity and membrane blebbing. **(G)** LDH release and **(H)** caspase-1 activity of cells under the different treatments. **(I)** The control, 4-OHT, -caffeine, -2-APB, -dantrolene-treated U87 cell monolayers were stained for β-actin (FITC), α-tubulin (TRITC). The images were generated from the fluorescence inverted microscope after immunofluorescence staining. **(J)** IHC analysis of caspase-1 expression in brain section of the sepsis mouse models. The cytoplasm was stained with the caspase-1 antibody (brown). Semiquantitative analysis of the IHC staining by AOD among the groups. Mean of ≥ 3 experiments ± SEM. Values marked with asterisks are significantly different, as determined using a *t*-test.

As previously mentioned, the pyroptosis process is accompanied by increased Ca^2+^ in astrocytes; and it is widely accepted that the calcium signal pathways are affected in response to pyroptosis (38, 39). To investigate the mechanisms by which intracellular Ca^2+^ regulates pyroptosis, the calcium release activator, caffeine (2 mM), and the inhibitors 2-APB (100 µM) and dantrolene (Dan) (20 µM) were employed. Regarding the cytoplasmic OP and PN count rates, caffeine treatment induced a statistically significant increase compared with that in the pyroptotic astrocyte control; whereas 2-APB or dantrolene treatment induced a decrease (**Figures 6A, B** and **Figures S6A, B**). Consistently, IF tensions were observed to correlate positively with the intracellular Ca^2+^ levels. Intriguingly, 2-APB could decrease the IF tension to control levels (**Figure 6C**). These data indicated that intracellular Ca^2+^ could regulate 4-OHT-induced cell osmolarity, IF tension upregulation, and the production inflammasome PNs. The intracellular Ca^2+^ and Cl^-^ levels were assessed using Fluo-4 AM and MQAE fluorescent imaging, respectively (**Figures 6D, E**), the results of which were coincident with the changes in cell osmolarity and IF tension.

Regarding to cell morphology, 4-OHT or LPS-NIG cotreated with caffeine promoted cell swelling and permeabilization; however, 2-APB or dantrolene presented the completely opposite morphology, showing a reduced number of swollen cells (**Figure 6F** and **Figure S6E**). LDH release and caspase-1 activity were upregulated when Ca^2+^ levels increased and downregulated when Ca^2+^ levels decreased (**Figures 6G, H** and **Figures S6D, E**). Interestingly, 4-OHT changed the morphological phenotypes of the cytoskeleton: filiform MF and MT structures turned into punctiform granules; whereas 2-APB and dantrolene attenuated this cytoskeletal deformation (**Figure 6I**). In addition, IHC staining of mouse brain sections indicated that the caspase-1 expression was closely related to intracellular Ca^2+^ levels after 4-OHT stimulus (**Figure 6J**). Overall, the abundant Ca^2+^ released from the endoplasmic reticulum played a crucial role in the pyroptosis process, involving the regulation of inflammasome production, PN-OP, and astrocyte blebbing and permeabilization.

### GSDMD-NT Induced PN-OP-Dependent Cell Swelling and Blebbing Through Activation of Calcium and Caspase-1

**Figure 7:**
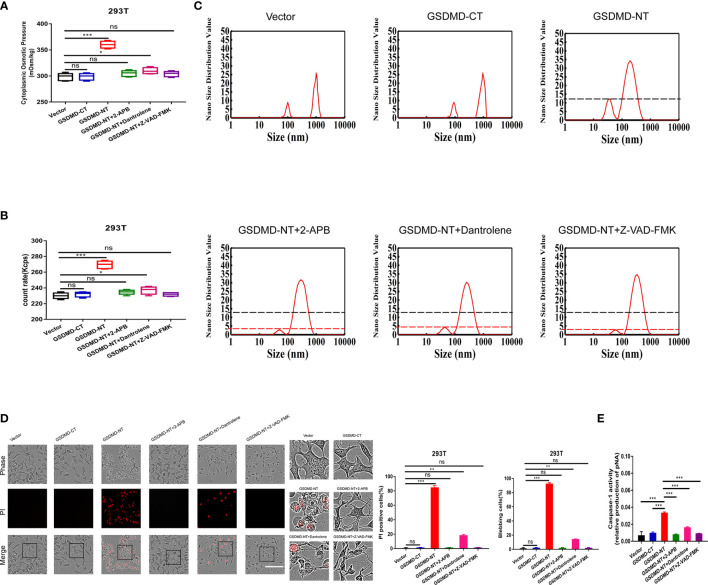
GSDMD-NT induced blebbing and permeabilization, regulated by Ca^2+^ and caspase-1 activation. **(A)** 293T cells were transfected individually with the vector control, GSDMD-CT, and GSDMD-NT. The cytoplasmic OP values of U87 cells were measured using a freezing point osmometer under the above stimuli. **(B)** The count rate of PNs in U87 cells. **(C)** Nano size distribution value of protein granules in the cytoplasm. **(D)** Representative time-lapse images of U87 cells under the different treatments. Cell membrane integrity was monitored by PI uptake (red fluorescence). The bar charts represent the percentage of cells showing PI positivity and blebbing. **(E)** Caspase-1 activity of cells. Mean of ≥ 3 experiments ± SEM. Values marked with asterisks are significantly different, as determined using a *t*-test. ns, not significant.

GSDMD-NT produced from GSDMD cleavage locates on the cell membrane, which is necessary to form membrane pores and induce permeabilization. GSDMD-NT and GSDMD-CT were individually over-expressed in human embryonic kidney 293T cells to determine their roles in regulating PN-OP during pyroptosis. Neither the vector control nor GSDMD-CT could change the cytoplasmic OP or PN numbers; however, GSDMD-NT over-expression resulted in a significant increase (**Figures 7A, B**). Compared with the 100 and 1000 nm sized PNs in the vector control and GSDMD-CT groups, the GSDMD-NT-induced PNs were mostly with distributed around 50-100 and 100-1000 nm, possibly resulting from NLRP3 PNs (**Figure 7C**). Consistent with previous reports, production of GSDMD-NT, rather than GSDMD-CT induced cell swelling, blebbing, membrane permeabilization and caspase-1 activity (**Figures 7D, E**). These data suggested that GSDMD-NT could facilitate NLRP3 production.

Additional 2-APB and dantrolene treatments were used separately to reduce the free Ca^2+^ levels in cells, and both treatments completely diminished the GSDMD-NT-induced increment in the cytoplasmic OP and PNs (**Figures 7A, B**), particularly for the small protein granules (50-100 nm PNs, **Figure 7C**). Morphological analysis also revealed that cell blebbing and permeabilization were significantly suppressed (**Figure 7D**), confirming that PN-OP in the pyroptosis process is regulated by the intracellular free Ca^2+^ levels. Interestingly, caspase-1 activity inhibited by Z-VAD-FMK led to a decrease in cytoplasmic OP, PNs and pyroptosis severity induced by GSDMD-NT over-expression (**Figures 7A–D**). This suggested that caspase-1 and calcium signaling are pivotal factors that regulate the swelling of pyroptotic cells. We speculated that Ca^2+^ and caspase-1 could play a role in controlling GSDMD-NT function, such as membrane location.

## Discussion

Astrocyte pyroptosis represents inflammatory cell necrosis, which is mediated by neuroinflammatory signaling, and dependents on caspase activation and GSDMD-NT-induced pore formation. The present study observed altered cytoskeleton tensions and osmotic differences across the cell membrane during the pyroptosis process. The mechanical mechanisms underlying pyroptosis are proposed: the production of inflammasomes and cytoskeleton depolymerization increase the amount of PNs and upregulate OP, thus promoting astrocyte swelling and blebbing and regulating the proptosis progression.

Inflammasomes could serve as PNs and predominantly account for the OP increase in astrocytic pyroptosis. Inhibition of NLRP3 assembly and activation effectively attenuated the increases in PN-OP and IF tension, suppressed astrocyte swelling and blebbing. In accordance with our results, Davis et al. have found that *ASC* or *NLRP3* knockout could not occur cell swelling; however, *CASPASE-1* knockout presented swollen cells (37). These suggest NLRP3 inflammasome mainly responsible for the PN-OP-induced cell deformation during pyroptosis, rather than the NLRP3 downstream events. In addition, we found that depolymerization of MF and MT to produce actin and tubulin, as another source of intracellular PNs that regulate OP. However, it seems to be the accompanied by other events. The phenomenon of astrocyte swelling induced by the increased PN-OP is under different mechanisms between cytotoxic brain edema and neuroglia pyroptosis. The astrocyte swelling caused by ischemia results in severe cytoskeleton depolymerization, which produces a mass of PNs to upregulate OP and cell edema *via* water influx (8). In contrast, cytoskeleton stabilization slightly reduces OP but does not affect membrane permeabilization in the astrocytic pyroptosis process. Previous research suggested that caspase-1 cleaves MT and mediates Shingshot-induced cofilin activation, which lead to MF depolymerization (16, 40). Therefore, PN-OP regulation during pyroptosis mostly depends on inflammasome PNs, but also on the accompanying cytoskeleton-depolymerized PNs in astrocytes.

PN-OP involvement in regulating pyroptotic astrocyte swelling can be partly explained by the Donnan effect (41–44). Inflammasomes with negative charges can adsorb abundant cations to form a compressed group. Notably, intracellular PN-ion adsorption disrupts the electrochemical equilibrium between two sides of the plasma membrane. To balance this, voltage-gated ion channels are activated and extracellular cations and anions are driven into cells, contributing to intracellular hyper-osmolarity and forming a new electrochemical equilibrium across the membrane. Furthermore, the intracellular Ca^2+^ increase might interfere with OP regulation *via* the release of PN-ion adsorption. According to the theory of double layers, divalent ions (Ca^2+^) can replace monovalent ions (mainly intracellular K^+^) when adsorbed by PNs, which rearranges PN-ion adsorption and regulates the effects of OP (45–47).

The present study proposes that inflammasome-induced OP increment is necessary for astrocyte swelling and blebbing, but is not absolutely required for membrane permeabilization. These are consistent with the previous report that *NLRP3* knock-out appeared not to cause swelling, and *GSDMD* knock-out showed swelling and blebbing, but not permeabilization (37). It is possible that the PN-induced hyperosmolarity facilitates blebbing to adjust the cell volume and prevent from the premature rupture. During the pyroptosis-induced cell deformation, the deformable membrane coupled with rigid cytoskeleton codetermine cell swelling and blebbing, which requires cytoskeleton remodeling and cytoskeletal tractive forces against outward osmotic force (9, 48–50). Intermediate filaments (IF), presenting the electric matrix within the cytosol, paly the key roles in maintaining cell shape. Using the biocompatible IF tension sensor of vimentin FRET-based probe, our study has found increase in the mechanically tractive force of IF with time during pyroptosis. Previous report observed that reduction in the inward MF tension facilitates production of larger blebs, while vanish of MT tension inhibits membrane blebbing (9). We speculate that the particular region on the membrane is disconnected from cytoskeletal network, NLRP3 inflammasome-induced hyperosmolarity could trigger the membrane extrusion to form blebs. Therefore, the early stage of astrocytic pyroptosis permits cell deformation of swelling and blebbing rather than the direct membrane rupture. Cells struggle against the osmotic difference across membrane, and finally permit the massive influx of non-selective ions and water induced by GSDMD pores, ultimately resulting in high permeabilization and cell disintegration. In addition, GSDMD-NT promotes the occurrence of pyroptosis, accompanied by the further generation of NLRP3 inflammasomes and the resultant PN-OP upregulation, suggesting that the increased number of PNs, but not non-selective ion influx, contributes to intracellular OP upregulation.

Interestingly, caspase-1, Ca^2+^ and NLRP3 inflammasomes can mutually regulate membrane blebbing and permeabilization in astrocytes. The present study found that caspase-1 inhibition dramatically decreased PN amount and the NLRP3 inflammasome levels, suggesting the existence of caspase-1-mediated feedback promotion of NLRP3 production. Previous research revealed that Ca^2+^ can control the activation of NLRP3 inflammasomes and further activate caspase-1-mediated pyroptosis (20, 51), which is supported by the present results showing that the low Ca^2+^ levels decreased caspase-1 activity. However, when the caspase-1 inhibitor was used, the Ca^2+^ levels were reduced in turn. It is possible that caspase-1 can also regulate NLRP3 activation by controlling the intracellular Ca^2+^ levels. Beyond the canonical relationship of NLRP3-caspase-1 (52, 53), neuroinflammation signaling seems to display the cascade-amplified roles that control the number of intracellular PNs in astrocytes.

In addition, GSDMD-NT-mediated pore formation appears to be regulated. Activated caspase-1 cleaved GSDMD to produce GSDMD-NT, which is located on the cell membrane and contributes to pore formation-mediated pyroptosis (35). When the GSDMD-NT was over-expressed and calcium ion or caspase-1 was inhibited at the same time, it was surprising that pyroptosis was blocked. This suggested that the location of GSDMD-NT on the membrane is dependent on intracellular calcium signaling and caspase-1 activation.

Pyroptosis of astrocytes mediates the inflammatory response in nervous system diseases. This study revealed the roles of NLRP3 inflammasomes, acting as PNs, in regulating the tension activity of astrocyte pyroptosis. The mutual relationships among swelling, blebbing and membrane permeabilization were further clarified for astrocytes. The inflammasome cascade produces massive amount of intracellular PNs, which effectively increase OP and promote swelling and blebbing. The accompanying increase in Ca^2+^ is involved in OP regulation, NLRP3 assembly, and GSDMD-NT-dependent pore formation. OP regulation of astrocyte pyroptosis provides new perspectives for mechanistic research.

## Data Availability Statement

The original contributions presented in the study are included in the article/**Supplementary Material**. Further inquiries can be directed to the corresponding author.

## Ethics Statement

The animal study was reviewed and approved by the Model Animal Research Center of Nanjing University of Chinese Medicine.

## Author Contributions 

Conceptualization, JG. Methodology, ZZ, TW, JC, HQ, CZ, WL, and SQ. Validation, ZZ and JG. Formal analysis, ZZ, TW, JT, and JG. Resources, JG. Data curation, ZZ, TW, and JG. Writing-original draft preparation, ZZ and TW. Writing-review and editing, ZZ and JG. Supervision, JG. Project administration, ZZ and JG. Funding acquisition, ZZ and JG. All authors contributed to the article and approved the submitted version.

## Funding

This research was funded by Grants from National Natural Science Foundation of China (No. 82073826), Key Program of Natural Science Foundation of Jiangsu Province (No. 19KJA320003), Natural Science Foundation of Jiangsu Province (No. BK20200844) and a Project Funded by the Priority Academic Program Development of Jiangsu Higher Education Institutions (Integration of Traditional Chinese and Western Medicine).

## Conflict of Interest

The authors declare that the research was conducted in the absence of any commercial or financial relationships that could be construed as a potential conflict of interest.
