# Supplementary material for: Inflammasome-Induced Osmotic Pressure and the Mechanical Mechanisms Underlying Astrocytic Swelling and Membrane Blebbing in Pyroptosis
Source: Front Immunol. 2021 Jul 7;12:688674. doi: 10.3389/fimmu.2021.688674 (PMC8293990; doi:10.3389/fimmu.2021.688674)
Supplement: Supplementary file 1 [file DataSheet_1.pdf]

Supplementary Material

1 Supplementary materials and methods

Reagents, antibodies and small interfering RNA (siRNA) design

4-Hydroxytamoxifen was purchased from Aladdin (Shanghai, China). Jasplakinolide, taxol, caffeine were obtained from Abcam (Cambridge, UK). 2-Aminoethyl diphenylborinate, dantrolene, z-vad-fmk, SP600125, LPS, nigericin were purchased from MedChemExpress (New Jersey, USA). MCC950 were obtained from CSNpharm (USA). PEG8000, PF431396 and Sytox Green were obtained from Beyotime Biotechnology (Shanghai, China). Cantharidin was purchased from Sigma-Aldrich (Saint Louis, MO, USA). Rabbit anti-caspase-1, rabbit anti-ASC and rabbit anti-NLRP3 antibodies were obtained from Proteintech (Chicago, USA). Mouse anti-α-tubulin antibody was from Boster (BM1452, Wuhan, China). Mouse anti-β-actin was purchased from Cell Signaling Technology (Danvers, MA, USA). The siRNA targeting *ASC* and *CASPASE-1* was constructed by GenePhrama (Shanghai, China). The Flag-Gsdmd-NT, Flag-Gsdmd-CT plasmid were purchased from Addgene (Massachusetts, USA).

Cell culture

The human glioblastoma cell line U87 was obtained by the American Type Culture Collection (ATCC, Manassas, USA). Cell were cultured at 37 °C, 5%CO_2 with complete Dulbecco’s modified Eagle’s Medium (Invitrogen, New York, USA) containing 10% fetal bovine serum (Invitrogen), 100 units/ml penicillin (Invitrogen) and 100μg/ml streptomycin.

Animals

The principles of laboratory animal care were followed and all procedures were conducted according to the guidelines established by the National Institutes of Health. The study protocol was approved by the Research Animal Care Committee of Nanjing University of Chinese Medicine. Adult male C57BL/6 mice (15-20g, six weeks old) were obtained from the Model Animal Research Center of Nanjing University of Chinese Medicine.

Measurement of the cytoplasmic OP and the count rate of protein particles

Cells were cultured in Petri dishes (100 mm). When cells reached 90% confluence, medium supplemented with special drugs replaced the old medium. Cells were washed with Hank’s isotonic solution three times and then trypsinized. Hank’s isotonic solution and trypsin had been adjusted to 290-310 mOsm/kg. The cell suspension was transferred into 1.5ml EP tube and centrifugated at 12000×g for 5 mins at 4°C. The supernatant was discarded. After ultrasonication (Sonics and Materials, Connecticut, CT, USA) at 5 s, five times, the cells were centrifuged at 4°C and 12000×g for 10 min

and 50 μl of the supernatant was moved to a new EP tube. The osmotic pressure and distribution of cytoplasmic nanoparticles were determined using freezing point osmometer and Nanosight NS300 instrument, respectively (Malvern Analytical, Malvern, UK)

Probe construction and transfection

Tension sensors were produced using the NovoRec PCR Seamless Cloning Kit and restriction enzyme cloning techniques according to previous reports (24-27). We constructed fluorescent sensors with circularly permutated cpVenus and cpCerulean (cpVenus–7aa–cpCerulean [cpstFRET]). The vimentin probe comprised PCMV-Vimentin-cpCerulean-7aa-cpVenus (cpstFRET)-Vimentin. In cpstFRET, cpCerulean (cyan) is the donor, and cpVenus (yellow) is the acceptor, and they are parallel under normal conditions. When the tension probe received an external force, the angle of cpstFRET changed and the FRET efficiency decreased. E.Z.N.A™ Endo-free Plasmid DNA Mini Kit II (Omega Bio-Tek, Norcross, GA, USA) was used to extract single-colony plasmids in accordance with the manufacturer instructions. We verified the integrity of all expression constructs using DNA sequencing.

cpstFRET analyses

The dipole angle between donor/enhanced cyan fluorescent protein (eCFP) and acceptor/enhanced yellow fluorescent protein (eYFP) determined the effectiveness of FRET. Cells were imaged using a confocal microscope (SP8; Leica) equipped with a ×63 oil-immersion objective lens. The donor and acceptor were tested by argon lasers at 458 nm and 514 nm, respectively. The CFP/FRET ratios were calculated using the equation 1/E = cerulean donor/venus acceptor.

FRET-AB and FRAP analyses

We applied LAS AF Application Wizard v1.7.0 (Leica) for detailed analyses of probes, including live cell acceptor photobleaching FRET (FRET-AB) experiments and fluorescence recovery after photobleaching (FRAP) experiments. The acceptor of the whole cell was bleached and then we calculated the efficiency of FRET. The constructed recovery curve was used to estimate probe activities.

Calcium and chloride fluorescence imaging

Fluo-4 AM was diluted to the working concentration (4 μM) using Hank's balanced salt solution (HBSS). Cells cultivated on confocal dishes were incubated in the Fluo-4 AM working solution for 20 min. After incubation, cells were added with HBSS with 1% fetal calf serum (five times the volume), and incubated for 40 min in an incubator. The cells were washed three times with the HBSS and then incubated at 37 ℃ for 10 min. Calcium ion fluorescence was detected using Thunder Imager (SP8; Leica) at excitation and emission wavelengths of 494 nm and 516 nm. The use of N-[ethoxycarbonylmethyl]-6-methoxy-quinolinium bromide (MQAE) was similar to Fluo-4 AM, except that the buffer was K-Hepes. Chloride ion fluorescence was detected at excitation and emission

wavelengths of 355 nm and 460 nm, respectively. The increase of intracellular Cl^- levels led to the decrease of MQAE fluorescence value instead.

Caspase-1 activity

Caspase-1 activity of U87 cells was assessed as per manufacturer's instructions (Beyotime Biotechnology, Shanghai, China). After cell suspension lysed on ice for 30 min, the PNA provided in the kit was diluted into a series of concentration gradient standard solutions, and the determination system was prepared according to the ratio of 1:9. The standard curve was obtained. The buffer system was prepared according to the kit, and the absorbance was measured at 405 nm by enzyme reader.

Immunohistochemistry (IHC) staining and analysis

Mouse brains were immersion-fixed in 4% paraformaldehyde solution at 4℃ for 24 h, dehydrated with a graded series of ethanol and xylene, embedded in paraffin, and cut into 5 μm thick sections. Brain tissues were stained using antibodies for caspase-1. The images were obtained at ×40 magnification under an inverted microscope (DMi8; Leica). Semi-quantitative analysis of the IHC images was conducted using Image J, in which the integral optical density (IOD) and the area data were collected. Then, the average optical density (AOD) was calculated as IOD/area, which represented the staining intensity.

Immunofluorescence

For immunocytochemical studies, cells were washed with room temperature phosphate-buffered saline (PBS) three times, fixed in 4% paraformaldehyde solution for 30 min, and washed again with PBS. The cell membrane was permeabilized in 0.1% Triton X-100 at 4℃ for 10 min. Cells were blocked with PBS containing 4% serum for 30 min at room temperature and then incubated with primary antibodies overnight at 4 ℃. After washing three times with PBS, cells were incubated with the secondary antibodies in the dark. DAPI was then used to stain the cell nuclei. Immunofluorescent staining was examined using a Leica confocal microscope (DMi8; Leica).

Statistical analyses

For quantitation, the ratio of eYFP/eCFP was measured using the Image J software. The FRET ratio in each subcellular region was measured for each cell and then averaged over multiple cells. Images were processed and pseudo-colored using the 16-color map of Image J. All data were presented as mean ± SEM. Single-factor analysis of variance (one way ANOVA) with the least significant difference test was adopted to determine statistical significance. Each experiment was repeated at least three times, >10 cells were imaged, and each condition was analyzed. P value were determined by t-test. Ns (not significant), $p > 0.05$, $*p < 0.05$, $**p < 0.01$, $***p < 0.001$.

2 Supplementary Figures

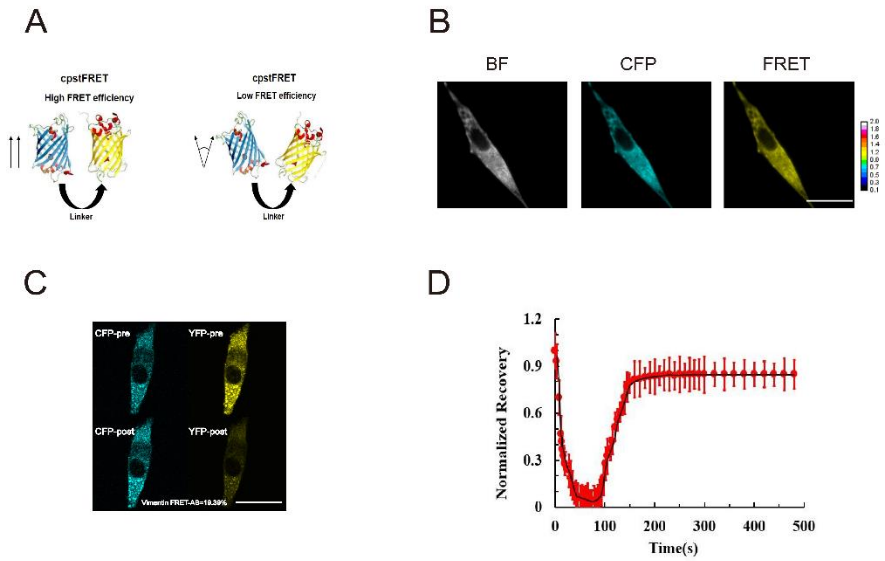

Figure S1. Construction and efficacy of the Vimentin probe in U87 cells. (A) U87 cells with the Vimentin probe were tested using 458 nm and 514 nm argon lasers. The Vimentin probe comprised PMVC-Vimentin-cpCerulean-7aa-cpVenus (cpstFRET)-Vimentin. CpVenus was parallel to cpCerulean in resting cells. When the external force across cpstFRET generated a certain angle, the Förster resonance energy transfer (FRET) efficiency was reduced (f: external force). (B) The CFP and YFP fluorescence signals of the Vimentin probe were analyzed under an inverted fluorescence microscope. (C) FRET acceptor photobleaching (AB) tested the reliability of the Vimentin probe. (D) The intracellular mobility of the Vimentin probe was examined using fluorescence recovery after photobleaching. The normalized average fluorescence recovery of Vimentin *vs*. time (500 s) was calculated. The fluorescence recovery ratio was noted. The calibration bar was set from 0.1 to 2.0. Scale bar, 10 μm, Mean of ≥ 3 experiments ± SEM.

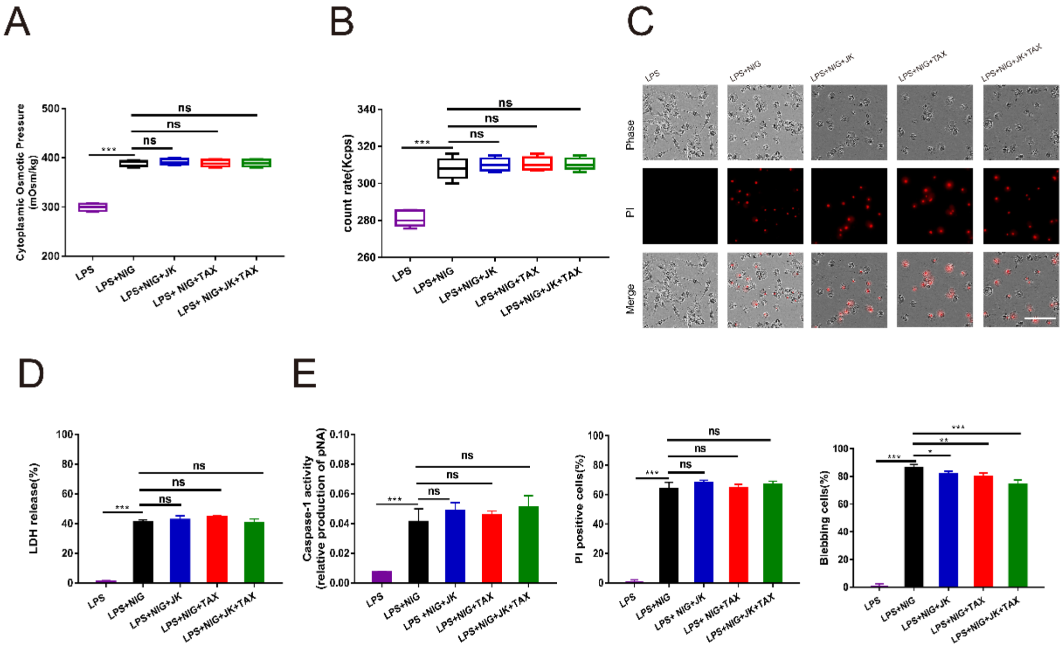

Figure S2. The LPS-NIG pyroptotic model co-treated with the MF and MT stabilizers. (A) The cytoplasmic OP values of U87 cells were measured using a freezing point osmometer under LPS-NIG treatment (LPS 1 μg/ml and NIG 20 μM), and co-treatments of LPS-NIG with JK, TAX, both agents, and the isotonic control. (B) The count rate of PNs in U87 cells. (C) Representative time-lapse images of U87 cells subjected to different treatments. Cell membrane permeabilization was monitored by PI uptake (red fluorescence). The bar charts represent the percentage of cells with propidium iodide (PI) positivity and blebbing. Scale bar, 100 μm. (D) LDH release and (E) caspase-1 activity of cells under the different treatments. Mean of ≥ 3 experiments ± SEM. Values marked with asterisks are significantly different, as determined using a *t*-test.

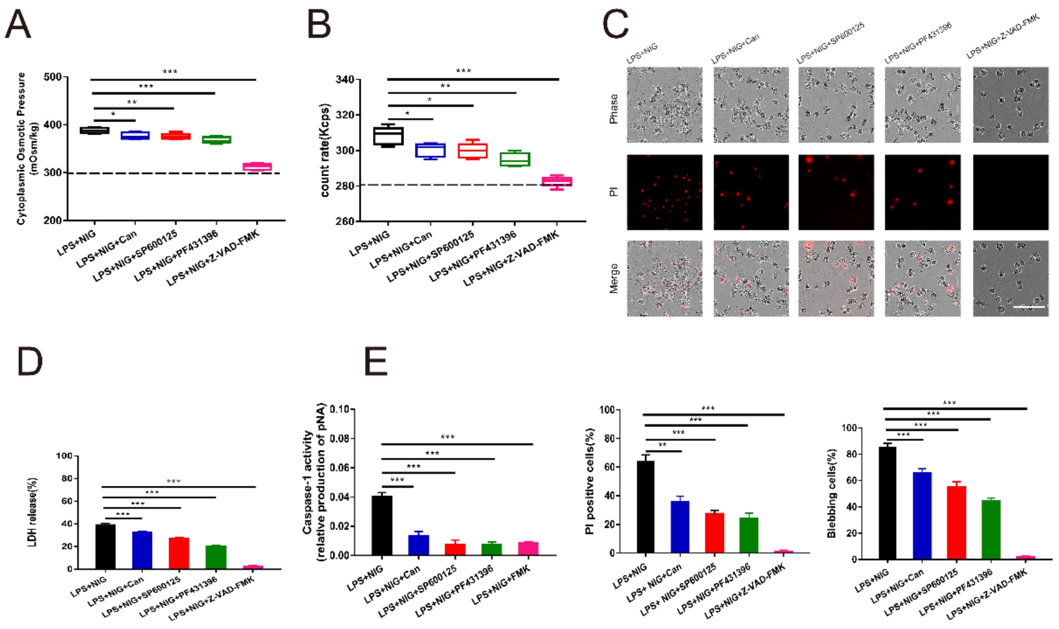

Figure S3. NLRP3 and caspase-1 inhibitors were employed with LPS-NIG pyroptotic astrocytes. (A) The cytoplasmic OP values of U87 cells were measured using a freezing point osmometer under LPS-NIG treatment, and co-treatments of LPS-NIG with Z-VAD-FMK, cantharidin, SP600125, and PF431396. (B) The count rate of PNs in U87 cells. (C) Representative time-lapse images of U87 cells subjected to different treatments. Cell membrane permeabilization was monitored by PI uptake (red fluorescence). Scale bar, 100 μm. The bar charts represent the percentage of cells with PI positivity and blebbing. (D) LDH release and (E) caspase-1 activity of cells under the different treatments. Mean of ≥ 3 experiments ± SEM. Values marked with asterisks are significantly different, as determined using a *t*-test.

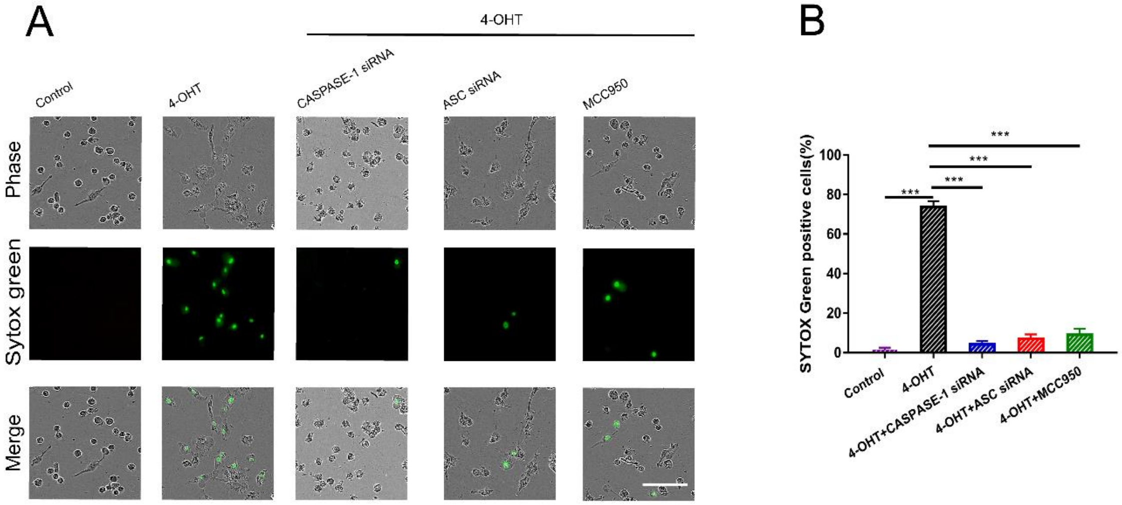

Figure S4. Membrane permeabilization monitored by SYTOX GREEN uptake. (A) Representative time-lapse images of U87 cells subjected to control, 4-OHT, both treatments of 4-OHT and *CASPASE-1* siRNA, *ASC* siRNA, or NLRP3 inhibitor MCC950. (B) The bar charts represent the percentage of SYTOX GREEN positive cells. Scale bar, 100 μm.

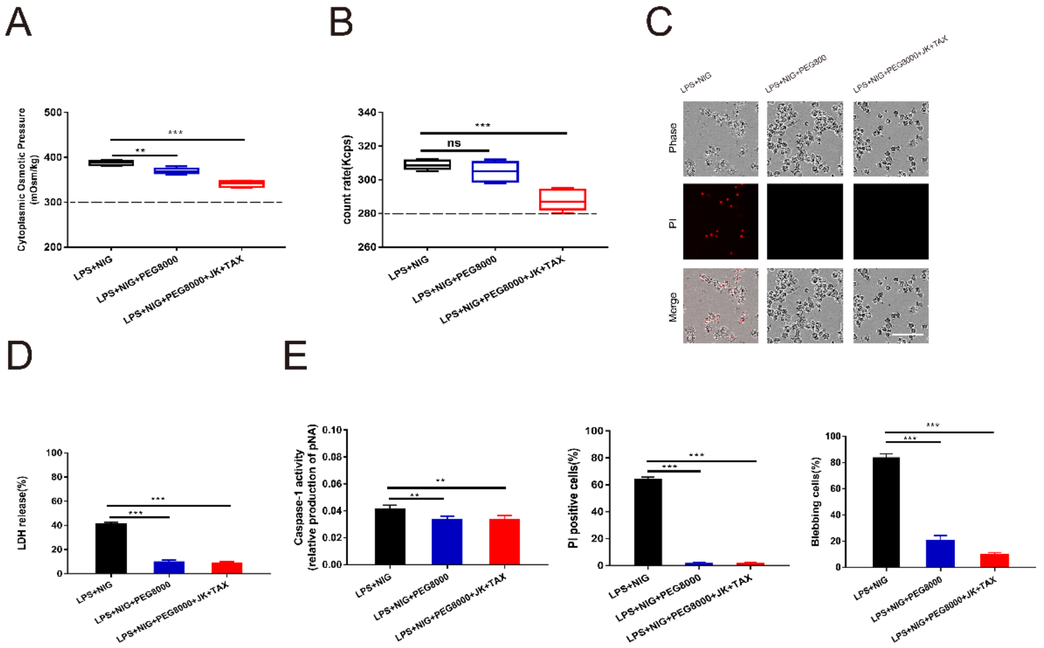

Figure S5. Blockage of non-selective membrane pores in the LPS-NIG model of pyroptotic astrocytes. (A) The cytoplasmic OP values of U87 cells were measured using a freezing point osmometer under LPS-NIG treatment, and co-treatments of LPS-NIG with PEG8000 and MF/MT stabilizers. (B) The count rate of PNs in U87 cells. (C) Representative time-lapse images of U87 cells subjected to different treatments. Cell membrane permeabilization was monitored by PI uptake (red fluorescence). Scale bar, 100 μm. The bar charts represent the percentage of cells showing PI positivity and blebbing. (D) LDH release and (E) caspase-1 activity of cells under the different treatments. Mean of ≥ 3 experiments ± SEM. Values marked with asterisks are significantly different, as determined using a *t*-test.

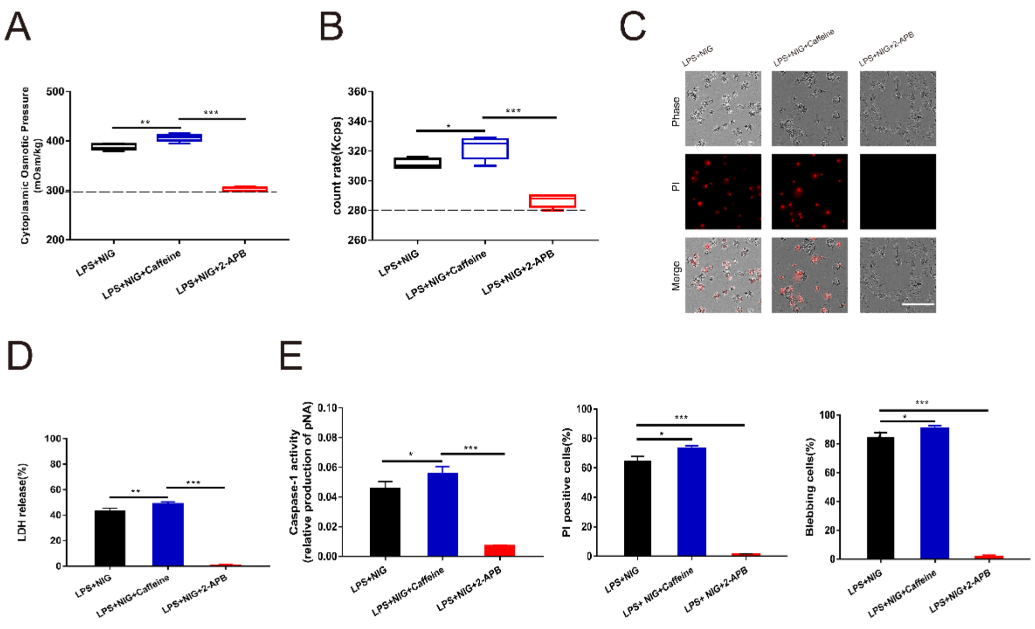

Figure S6. Manipulation of the intracellular Ca^{2+} level in the LPS-NIG model of pyroptotic astrocytes. (A) The cytoplasmic OP values of U87 cells were measured using a freezing point osmometer under LPS-NIG treatment, and co-treatments of LPS-NIG with Caffeine and 2-APB. (B) The count rate of PNs in U87 cells. (C) Representative time-lapse images of U87 cells subjected to different treatments. Cell membrane permeabilization was monitored by PI uptake (red fluorescence). Scale bar, 100 μm. The bar charts represent the PI positive and blebbing cells. (D) LDH release and (E) caspase-1 activity of the cells. Mean of ≥ 3 experiments ± SEM. Values marked with asterisks are significantly different, as determined using a *t*-test.
